# Prevalence of the Metabolic Syndrome and its determinants among Nepalese adults: Findings from a nationally representative cross-sectional study

**DOI:** 10.1038/s41598-018-33177-5

**Published:** 2018-10-09

**Authors:** Suresh Mehata, Nipun Shrestha, Ranju Kumari Mehta, Bihungum Bista, Achyut Raj Pandey, Shiva Raj Mishra

**Affiliations:** 1 Ipas Nepal, Kathmandu, 44600 Nepal; 2 Institute for Health and Sport (IHES), Victoria University, Melbourne, 1300 VIC UNI Australia; 3 Nepal Health Research Council, Ministry of Health and Population, Kathmandu, 44600 Nepal; 4 Nepal Development Society, Chitwan, 44207 Nepal; 5 Division of Epidemiology and Biostatistics, Faculty of Medicine, University of Queensland, Brisbane, 4072 Australia

## Abstract

Metabolic syndrome (MetS) increases the risk of cardiovascular diseases and diabetes mellitus. This study is designed to assess the prevalence and determinants of MetS among Nepalese adults from a nationally representative study. This study is based on Stepwise Approach to Surveillance (STEPS) Survey from Nepal. This survey was done among 4200 adults aged 15–69 years from 210 clusters selected proportionately across Nepal’s three ecological zones (Mountain, Hill and Terai). Subsequently, using systematic sampling, twenty households per cluster and one participant per household were selected. The overall prevalence of MetS is 15% and 16% according to Adult Treatment Panel III (ATP III) and International Diabetes Federation (IDF) criteria respectively. A triad of low HDL-C, abdominal obesity and high BP was the most prevalent (8.18%), followed by abdominal obesity, low HDL-C cholesterol and high triglycerides (8%). Less than two percent of participants had all the five components of the syndrome and 19% of participants had none. The prevalence steadily rose across the age group with adults aged 45–69 years having the highest prevalence (28–30%) and comparable prevalence across two definitions of MetS. A notably high burden for females, urban, hill or Terai resident were seen among other factors.

## Introduction

The inter-related cluster of cardio-metabolic risk factors comprising of elevated fasting glucose, elevated blood pressure, elevated triglycerides (TG), reduced high-density lipoprotein (HDL) and central obesity has been termed Metabolic syndrome (MetS) in the literature^1^. There is two fold increase in risk for cardiovascular disease and five fold increase in risk for type II diabetes in people with MetS compared to those without syndrome^2,3^. Hence treatment and prevention of MetS is of paramount importance as a means of lowering the risk of diabetes and cardiovascular disease (CVD).

There is large variation in global prevalence of MetS ranging from 7.1% to 41.6% across studies due to lack of consensus definition of MetS^4–6^. The most commonly used criteria are the International Diabetes Federation adopted in 2005 (IDF)^7^ and Adult Treatment Panel III (ATPIII) adopted in 2005^4^. Although the mechanism of MetS has not been elucidated fully, various factors such as unhealthy diet, physical inactivity, urbanization and underlying genetic predisposition have been found to be associated with MetS^8^.

With rapid demographic and epidemiologic transition in Nepal, the burden of non-communicable diseases (NCDs), notably cardiovascular disease (CVD) has escalated to epidemic proportion^9^. A recent hospital based study conducted in a random sample of indoor patients from tertiary levels hospitals found that 31% of admitted patients suffer from NCDs; with cardiovascular disease and diabetes accounting for 40% and 12% of NCDs respectively^10^. This hospital based study might not reflect the true prevalence of NCDs at population level in Nepal as utilization of health services remains poor and urban-centric. The absence of routine surveillance and registry system in Nepal has further made it challenging to precisely estimate the burden of MetS^11^. An earlier prevalence study limited to eastern region of Nepal found one in five Nepalese adults have MetS^12^. This is comparable to studies from neighboring South Asian countries like India, Pakistan and Bangladesh where nearly a third of population have MetS^13,14^. None of the studies conducted thus far assessed the attributing factors in occurrence of MetS in Nepal.

The unavailability of nationally representative prevalence estimates and factors attributing on MetS has hindered the development of targeted strategies against MetS. A focus on individual components of MetS is a norm in routine practice, as well as the recently formed Multi-sectoral Action Plan of NCDs from Ministry of Health of Nepal addresses hypertension, diabetes, obesity and raised cholesterol separately while MetS is unaddressed giving the lack of evidence at population level. Hence, it is important to investigate the prevalence and determinants of MetS at entire-population level, using the nationally representative data. This is important for local planning, addressing the population burden via concerted actions on its determinants and targeting the high-risk groups.

## Results

This secondary-analysis of the data provided by the nationally representive survey (STEPS Survey), provides the first nationally representative prevalence of MetS among adult population of Nepal. A total of 3729 participants aged 15 to 69 years were assessed for physical and biochemical parameters. The prevalence of metabolic syndrome is 15% and 16% according to ATP III and IDF criteria respectively. Overall, 21% of participants had had three or more risk factors. The most predominant component of MetS in this population was low HDL cholesterol (71%), followed by high blood pressure (26%) and raised triglycerides (25%). A significantly high prevalence of abdominal obesity and low HDL cholesterol was observed among female participants; whereas raised triglycerides, fasting blood sugar and high blood pressure was observed among male participants (Table 1).

**Table 1:** Frequency distribution of various combinations of components of metabolic syndrome among the participants (N = 3729).

	Both sexes (N = 3729)	Male (N = 1190)	Female (N = 2539)	P
Abdominal obesity*	27.47	18.03	36.49	<0.001
Raised triglycerides (TRI)^ɫ^	25.26	31.78	19.50	<0.001
Low HDL cholesterol^ǂ^	70.72	61.22	79.29	<0.001
Raised fasting blood glucose^§^	17.59	21.36	13.71	<0.001
High blood pressure^¶^	26.28	30.84	20.18	<0.001
0 or <3 components	78.85	79.03	78.67	0.923
Low HDL + TRI + high BP	6.90	6.41	9.18	0.014
Low HDL + TRI + abdominal obesity	7.83	1.04	5.28	0.011
Low HDL + TRI + abdominal obesity + high BP	3.92	3.91	3.93	0.977
Low HDL + TRI + abdominal obesity + high BP + FG	1.30	0.96	1.64	0.007
Low HDL + abdominal obesity + high BP	8.18	6.85	9.45	0.010
Low HDL + abdominal obesity + FG	4.74	3.40	6.02	0.003
TRI + abdominal obesity + high BP + FG	1.82	1.72	1.92	0.647
Abdominal obesity + FG + High BP	3.62	3.71	3.52	0.768

^*^Abdominal obesity (waist ≥90 cm in men or ≥80 cm in women).

^ɫ^Raised triglycerides (≥150 mg/dL or specific treatment for this lipid abnormality).

^ǂ^Low HDL cholesterol (<40 mg/dL in men; <50 mg/dL in women or specific treatment for this lipid abnormality).

^§^Raised fasting blood glucose (≥100 mg/dL or previously diagnosed type 2 diabetes).

^¶^High BP (systolic BP ≥130 or diastolic BP ≥85 mm Hg or treatment of previously diagnosed hypertension).

BMI, body mass index; BP, blood pressure; HDL, high-density lipoprotein.

**Figure 1:**
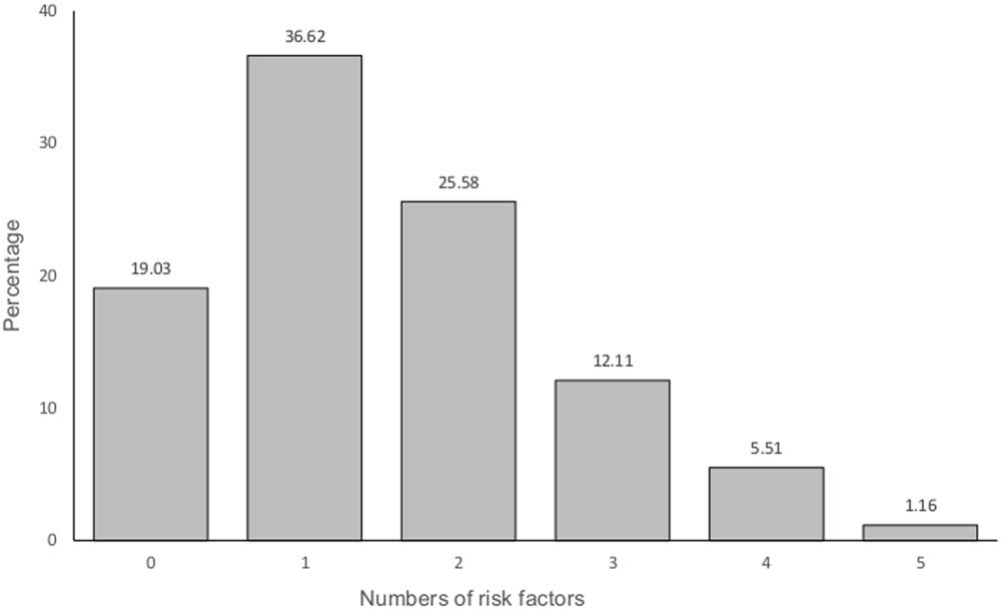
Prevalence of various components of metabolic syndrome among participants aged 15–69 years.

A triad of low HDL-C, abdominal obesity and high BP was the most prevalent constituting 8.2% of the total participants, followed by abdominal obesity, low HDL-C cholesterol and high triglycerides at 8% of participants. Less than two percent of participants had all the five components of the syndrome (Table 1). Only 19% of participants had zero risk factors (Fig. 1).

Table 2 presents the independent effect of various covariates on the clustering of MetS components at the individual level using multivariate Poisson regression. The age, education, caste/ethnicity, abnormal waist hip ratio, BMI and place of residence were independently associated with the number of MetS components. Moreover, the cumulative risk of having x number of MetS risk factors or more versus having fewer were 1.28 times higher among participants aged 30–44 years and 1.52 times higher among participants aged 45–69 years compared to 15–29 years. The cumulative risks increased with increase in age and educational level. Participants those who resides in urban areas were 1.13 times more likely to have x or more risk factors compared to rural residence.

**Table 2:** Mean number various components of metabolic syndrome and independent effects of covariates on metabolic syndrome components clustering in individuals.

Age group	Un-weighted	Mean number of risk factors (95%CI)	ARR*^a^	P
15–29	831	1.28 (1.20–1.36)	1	
30–44	1,410	1.85 (1.76–1.94)	1.28 (1.19–1.38)	<0.001
45–69	1,488	2.14 (2.04–2.24)	1.52 (1.41–1.63)	<0.001
**Gender**				
Male	1,190	1.64 (1.54–1.74)	1	
Female	2,539	1.71 (1.64–1.77)	1.03 (0.98–1.09)	0.247
**Education**				
No formal schooling	1,662	1.77 (1.68–1.86)	1	
Primary	917	1.67 (1.56–1.78)	1.03 (0.97–1.09)	0.325
Secondary	705	1.60 (1.47–1.74)	1.07 (1.00–1.16)	0.057
Higher	445	1.61 (1.49–1.74)	1.12 (1.03–1.23)	0.011
**Marital status**				
Never married	299	1.24 (1.12–1.36)	1	
Currently married	3,222	1.77 (1.70–1.84)	1.00 (0.90–1.12)	0.950
Divorced/Widowed/Separated	207	1.79 (1.58–2.00)	0.89 (0.78–1.03)	0.111
**Caste/ethnicity**				
Dalits	317	1.76 (1.61–1.92)	1	
Disadvantaged Janajatis	1,165	1.60 (1.51–1.69)	0.91 (0.82–1.01)	0.069
Disadvantaged non-Dalit Terai caste groups	296	1.50 (1.33–1.66)	0.88 (0.77–0.99)	0.036
Religious minorities	46	2.00 (1.53–2.47)	1.06 (0.83–1.34)	0.640
Relatively advantaged Janajatis	287	1.88 (1.61–2.15)	0.88 (0.77–1.00)	0.050
Upper caste groups	1,618	1.70 (1.59–1.80)	0.88 (0.80–0.98)	0.015
**Ecological zone**				
Mountain	260	1.56 (1.30–1.82)	1	
Hill	1,568	1.76 (1.64–1.88)	0.99 (0.83–1.17)	0.881
Terai	1,901	1.62 (1.54–1.70)	0.98 (0.83–1.15)	0.798
**Place of residence**				
Rural	3,022	1.60 (1.53–1.67)	1	
Urban	707	1.99 (1.81–2.16)	1.13 (1.05–1.21)	0.001
**Current smoking**				
No	3,051	1.68 (1.61–1.76)	1	
Yes	678	1.63 (1.51–1.74)	0.96 (0.89–1.03)	0.244
**Harmful use of alcohol**				
No	3,663	1.67 (1.61–1.74)	1	
Yes	66	1.70 (1.30–2.10)	1.10 (0.87–1.40)	0.417
**Insufficient fruit and vegetable intake**				
No	43	1.78 (1.36–2.20)	1	
Yes	3,686	1.67 (1.60–1.74)	1.07 (0.93–1.23)	0.330
**Low physical activity**				
No	3,580	1.67 (1.60–1.73)	1	
Yes	133	1.90 (1.64–2.16)	1.04 (0.94–1.16)	0.419
**Abnormal waist hip ratio**				
No	1,056	1.29 (1.21–1.36)	1	
Yes	2,673	1.94 (1.86–2.02)	1.35 (1.27–1.43)	<0.001
**BMI category**				
<25	2,773	1.42 (1.35–1.48)	1	
25–30	763	2.50 (1.39–2.62)	1.51 (1.43–1.60)	<0.001
>30	193	2.95 (2.72–3.19)	1.65 (1.52–1.78)	<0.001
**Total**	**3,729**	1.67 (1.61–1.74)		

^*^The number of components of metabolic syndrome was the dependent variable. Each Relative Risk (RR) reflects the risk of having x or more components versus having fewer against the risk in the reference group. Hence, the Adjusted RR (ARR) represents the average effect of the covariate on the risk of having x number of components or more.

^a^ARR have been adjusted for all variables listed.

**Figure 2:**
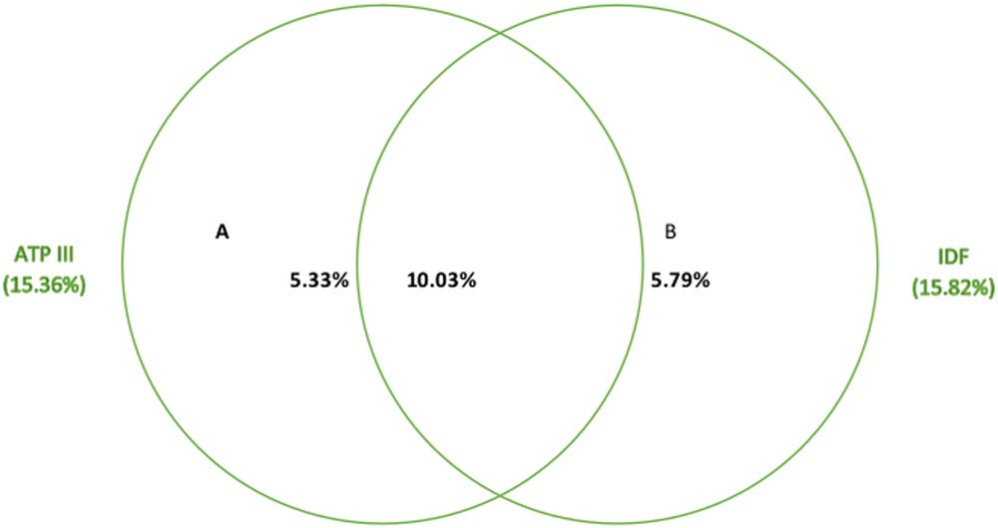
Prevalence of the metabolic syndrome according to ATP III and IDF definitions.

Table 3 presents prevalence of MetS assessed using ATP III and IDF criteria. The prevalence was higher among participants aged 45 to 69 years, those who do not have formal education, widowed/divorced/separated, belongs to religious minorities, those who resides in hilly region, and among urban dwellers. Likewise, the higher prevalence was also observed among participants who had abnormal waist hip ratio, and were obese [BMI ≥ 30 kg/m^2^]. The differences in the prevalence of the MetS using ATP III and IDF criteria can be demonstrated by data from the study. Although both the definition identified approximately 16% of the population as having the MetS, there was a large variability and only 10% of individuals met the criteria for both the definitions (Fig. 2).

**Table 3:** Prevalence of metabolic syndrome by demographic and clinical risk factors.

	Un-weighted	ATP III	IDF
**Age group**			
15–29	831	4.71	6.06
30–44	1,410	18.3	19.43
45–69	1,488	29.97	28.27
*P-value*		<0.001	<0.001
**Gender**			
Male	1,190	15.30	13.42
Female	2,539	15.43	18.10
*P-value*		0.923	0.002
**Level of education**			
No formal schooling	1,662	17.87	19.01
Primary	917	15.5	16.43
Secondary	705	15.39	14.31
Higher	445	11.05	11.80
*P-value*		0.021	<0.001
**Marital status**			
Never married	299	3.79	3.19
Currently married	3,222	17.83	18.66
Divorced/Widowed/Separated	207	22.52	19.99
*P-value*		<0.001	<0.001
**Caste/ethnicity**			
Dalits	317	15.31	17.11
Disadvantaged Janajatis	1,165	13.29	13.05
Disadvantaged non-Dalit Terai caste groups	296	10.99	12.39
Religious minorities	46	25.25	16.32
Relatively advantaged Janajatis	287	23.04	23.70
Upper caste groups	1,618	16.12	16.83
*P-value*		0.007	0.020
**Ecological zone**			
Mountain	260	12.45	5.56
Hill	1,568	19.09	18.36
Terai	1,901	12.65	14.96
*P-value*		0.002	0.001
**Place of residence**			
Rural	3,022	13.42	13.75
Urban	707	23.42	24.36
*P-value*		<0.001	<0.001
**Current smoking**			
No	3,051	15.68	16.69
Yes	678	13.99	12.00
*P-value*		0.405	0.012
**Harmful use of alcohol**			
No	3,663	15.25	15.96
Yes	66	20.72	9.23
*P-value*		<0.458	0.196
**Insufficient fruit and vegetable intake**			
No	43	12.84	17.83
Yes	3,686	15.39	15.79
*P-value*		0.665	0.786
**Low physical activity**			
No	3,580	15.04	15.71
Yes	133	24.95	18.87
*P-value*		0.011	0.403
**Abnormal waist hip ratio**			
No	1,056	7.3	2.94
Yes	2,673	20.93	24.70
*P-value*		<0.001	<0.001
**BMI category**			
<25	2,773	8.76	6.85
25-<30	763	33.83	43.84
≥30	193	61.2	64.71
*P-value*		<0.001	<0.001
**Total**	**3,729**	**15.36**	**15.82**

In adjusted model, age, gender, abnormal waist hip ratio, BMI, ecological zone, place of residence were the significant predictor of MetS by both the criteria (Table 4).

**Table 4:** Univariate and multivariate analyses of demographic and clinical risk factors for metabolic syndrome.

	ATP III Criteria				IDF Criteria			
	OR	95%CI	OR^#^	95%CI	OR	95%CI	OR^#^	95%CI
**Age group**								
15–29	1		1		1		1	
30–44	4.53	2.81–7.32**	3.04	1.84–5.03**	3.74	2.58–5.41**	2.07	1.31–3.25*
45–69	8.66	5.46–13.73**	7.08	4.26–11.78**	6.11	4.31–8.65**	4.52	2.91–7.03**
*Gender*								
*Male*	1				1		1	
*Female*	1.01	0.82–1.24	0.97	0.75–1.26	1.43	1.14–1.79*	1.44	1.05–1.97*
**Level of education**								
No formal schooling	1				1		1	
Primary	0.84	0.65–1.10	1.11	0.82–1.51	0.84	0.65–1.07	1.08	0.79–1.49
Secondary	0.84	0.62–1.13	1.53	1.07–2.19*	0.71	0.54–0.93*	1.11	0.76–1.63
Higher	0.57	0.41–0.80*	1.03	0.65–1.63	0.57	0.41–0.79*	0.90	0.57–1.41
**Marital status**								
Never married	1				1			
Currently married	5.5	2.37–12.79**	1.25	0.50–3.12	6.97	3.30–14.72**	1.72	0.73–4.08
Divorced/Widowed/Separated	7.37	2.98–18.21**	1.03	0.39–2.73	7.59	3.44–16.75**	1.12	0.44–2.85
**Caste/ethnicity**								
Dalits	1		1		1		1	
Disadvantaged Janajatis	0.85	0.55–1.31	0.79	0.48–1.33	0.73	0.46–1.14	0.58	0.34–1.01
Disadvantaged non-Dalit Terai caste groups	0.68	0.39–1.19	0.78	0.45–1.34	0.69	0.37–1.26	0.64	0.32–1.26
Religious minorities	1.87	0.87–4.01	1.99	0.76–5.18	0.94	0.33–2.74	0.65	0.16–2.63
Relatively advantaged Janajatis	1.66	0.97–2.84	0.73	0.42–1.28	1.50	0.88–2.59	0.65	0.36–1.19
Upper caste groups	1.06	0.70–1.61	0.76	0.48–1.20	0.98	0.63–1.52	0.61	0.36–1.06
**Ecological zone**								
Mountain	1		1		1		1	
Hill	1.66	0.89–3.10	1.04	0.49–2.18	3.82	2.00–7.31**	2.41	1.04–5.63*
Terai	1.02	0.56–1.87	0.75	0.36–1.55	2.99	1.57–5.70*	2.64	1.14–6.11*
**Place of residence**								
Rural	1		1		1		1	
Urban	1.97	1.44–2.71**	1.56	1.14–2.13*	2.02	1.48–2.76**	1.56	1.14–2.13*
**Current smoking**								
No	1				1			
Yes	0.87	0.64–1.20	0.76	0.51–1.13	0.68	0.50–0.92*	0.71	0.50–1.01
**Harmful use of alcohol**								
No	1		1		1		1	
Yes	1.45	0.54–3.93	1.80	0.46–7.03	0.54	0.20–1.41	0.69	0.21–2.28
**Insufficient fruit and vegetable intake**								
No	1		1		1		1	
Yes	1.24	0.47–3.23	2.21	0.75–6.50	0.86	0.30–2.49	1.24	0.42–3.63
**Low physical activity**								
No	1		1		1		1	
Yes	1.88	1.15–3.08*	1.48	0.89–2.47	1.25	0.74–2.10	0.93	0.54–1.59
**Abnormal waist hip ratio**								
No	1		1		1			
Yes	3.36	2.40–4.70**	2.50	1.79–3.50**	14.46	10.48–19.95**	8.18	5.93–11.29**
**BMI category**								
<25	1		1		1		1	
25–30	5.32	4.06–6.97**	3.74	2.79–5.02**	10.61	8.13–13.84**	7.82	5.78–10.78**
>30	16.42	10.84–24.87**	9.44	6.03–14.78**	24.92	16.02–38.77**	14.03	8.71–22.60**

^#^Adjusted relative risk have been adjusted for all variables listed.

*p < 0.05; **P < 0.001.

Using IDF criteria, compared to participants aged 15–29 years, the higher odds of occurrence of MetS was observed among participation aged 30–44 years (AOR: 2.07; 95% CI: 1.31–3.25) and 45–69 years (AOR: 4.52; 95% CI: 2.91–7.03). The odds of occurrence of MetS was significantly higher among female (AOR: 1.44; 95% CI: 1.05–1.97) compared to male counterparts. Likewise, participants who had abnormal waist hip ratio had higher likelihood of MetS compared to normal (AOR: 8.18; 95% CI: 5.93–11.29), and the study also revealed that the higher risk of occurrence of MetS among overweight (AOR: 7.82; 95% CI: 5.78–10.78) and obese (AOR: 14.03; 95% CI: 8.71–22.60) compared to those who had <25 kg/m^2^ BMI. Also, compared to participants who resides in mountains, the higher likelihood of occurrence among the residence who resides in Terai/the plain (AOR: 2.64; 95% CI: 1.14–6.11) and hill (AOR: 2.41; 95% CI: 1.04–5.63). The higher odds of occurrence of MetS was observed among urban dwellers (AOR: 1.56; 95% CI: 1.14–2.13) compared to rural dwellers.

## Discussion

This secondary-analysis of the data provided by the nationally represented survey (STEPS Survey), provides the first nationally representative estimates on prevalence, disaggregated by sub-groups, and factors attributed to MetS among adult population of Nepal. The overall prevalence of metabolic syndrome is 15% and 16% according to ATP III and IDF criteria respectively. Based on our findings, the Nepalese population appears to have a relatively lower burden of MetS compared to overall burden of MetS in South Asia (ATPIII 26.1% and IDF 29.8%) reported by a systematic review^15^. A triad of central obesity, low HDL-C, and elevated BP was the most prevalent (8.2%) combination of CVD risk factors constituting the syndrome in this population. This was followed by a triad of CVD risk factors, namely central obesity, low HDL-C and elevated triglycerides found in 7.8% of the total participants. The most common MetS component was low HDL cholesterol.

In a multivariable analysis, the risk of MetS increased steadily with age: participants aged 45–69 were 7.08 (ATP III) and 4.52 (IDF) times more likely to suffer from MetS than those who were in the age group 15–29 years. Similar findings were also seen with BMI: participants with BMI ≥ 30 kg/m^2^ were 9.44 (ATP III) and 14.03 (IDF) times more likely to suffer from MetS than those who had BMI < 25 kg/m^2^. The prevalence of MetS is higher in females compared to males in Nepal. The association with age, sex and BMI were consistent with previous studies from different countries^13,16,17^. People living in urban areas were twice more likely to develop MetS compared to rural residents. This might be due to sedentary lifestyles, dietary changes and stress in urban people.

The association of MetS with low physical activity was inconsistent with increased odds of 1.48 and decreased odds of 0.93 according to ATP III and IDP criteria respectively. However, some epidemiologic studies and uncontrolled trials have suggested that increased moderate-to-vigorous physical activity reduces the incidence or prevalence of the MetS^18–21^. Insufficient fruit and vegetable intake and cigarette smoking increased the odds of developing MetS in this study. Consumption of fruits and vegetables has been found to reduce diastolic blood pressure^22^ and risk of type II diabetes mellitus^23^ and may therefore reduce the risk of MetS. Current smoking was not-significantly associated with lower risk of MetS in our study. Existing evidence for association of MetS with cigarette smoking is inconsistent as well^24–26^. In our study, there was inconsistent evidence of association of alcohol intake and MetS with increased risk according to ATP III criteria and reduced risk according to IDP criteria. Various studies have shown that drinking alcohol is harmful at higher doses however, light to moderate doses is beneficial to health and reduces risk of coronary heart disease, diabetes, stroke and total mortality in adults^27–29^. A recent academic paper by international experts recommended lifestyle changes comprising of increased intake of fruits and vegetables, quitting smoking, moderate consumption of alcohol and physical activity to prevent MetS and improve cardio metabolic health^30^.

South Asians have a propensity towards less lean muscle mass and more visceral fat mass at a lower BMI and waist circumference compared to western population who tend to carry much of their weight in muscle and subcutaneous fat depots peripherally^31,32^. Thus South Asians with same body mass index are be at higher risk for metabolic syndrome compared to their western counterparts^33^. Moreover, comorbid diseases, including T2DM, occur at lower waist circumference in Asian adults^34^. Thus, ATP III might result in low prevalence figures in Nepalese population due to use of non-specific cut-offs for waist circumference. Moreover, waist circumference is used as only optimal component in ATP III. IDF criteria with waist circumference thresholds specific to Asian population (men 90 cm and women 80 cm) might be therefore more suitable for estimation of metabolic syndrome among adult Nepalese population.

Much of the health care priorities to date in low and middle income countries, including Nepal has been catering to infectious diseases and maternal and child health. A high burden of MetS as we demonstrated in our study and confirmed by other small studies from non-specialist hospital setting necessitates acknowledging the importance of reducing NCD risk factors at population level. The primary goal should be creating environment that facilitates greater physical activity and awareness regarding healthy dietary choices. In addition, the health systems need to be strengthened to reach the populations that are at risk for NCDs. Additionally, alternate models for service delivery and health promotion should be considered to reach unprivileged population with limited access to health care services. These may include mobilization of community health volunteers and mass media campaigns aimed at changing risky lifestyle behaviors.

Nationally, representative data are limited in Nepal and prevalence figures in the earlier studies were estimated using non-comparable sampling design in a section of population. This study is based on a large national sample consisting of both urban and rural populations in Nepal. However, as in any cross-sectional study, we could not establish causality between metabolic syndrome and its determinants. The South Asian population have been shown to be genetically susceptible to central obesity and insulin resistance^35^. However, the absence of genetic studies in Nepal have limited our understanding of the role of genetic factors in the pathogenesis of metabolic syndrome among Nepalese adults. The STEPS survey did not include information on household wealth or monthly income of participants hence an important determinant of metabolic syndrome could not be assessed among Nepalese population in this study.

## Conclusion

Our study demonstrates a high burden of MetS in Nepalese adults with ten percentage increment in prevalence across each age groups fifteen years apart. The prevalence peaked among those aged 45–69 years such as three in ten in this age-group had MetS. A substantial clustering of risk factors was evident with 80.7% having at least one risk factor. A triad of low HDL-C, abdominal obesity and high BP was the most prevalent (8.18%), followed by abdominal obesity, low HDL-C cholesterol and high triglycerides (8%). Female gender, urban, hill or terai resident were particularly at risk.

## Methods

### Study design and sampling technique

This is a secondary analysis carried out using Non-Communicable Diseases risk factors 2013 data. The detail methodology and report of the survey has been presented elsewhere^36^. In brief, the survey was a cross sectional study, carried out from January to June 2013, with aim to assesses the risk factors of NCDs at entire population level.

Firstly, out of the 921 Ilakas (an administrative unit at the sub-district level) in Nepal, 70 were selected, which were proportionately distributed across Nepal’s three ecological zones (Mountain, Hill and Terai/Plains) using probability proportionate to population size (PPS) which served as a primary sampling unit (PSU) of the study. Secondly, three clusters, the wards (the smallest administrative units) were selected from each of the PSU using the PPS. A total of 210 clusters were selected served as secondary sampling unit of this study. Subsequently, using systematic sampling, twenty households per cluster were selected. Using Kish method, one participant in each household were selected from the eligible frame (15–69 years). A sample size of 4,200 was used to represent the adult target population aged 15–69 years in Nepal. In order to calculate the required sample size the prevalence of low fruit and vegetable consumption was considered 61.9%, absolute margin of error ±5%, design effect 1.5, response rate 80% and six domains (three age categories and two genders) were considered to obtain nationally representative sample. A detail algorithms of sample size calculation have presented elsewhere^36^.

### Survey implementation

A total of 26 researchers divided into 2 teams were assigned for data collection. Each team was composed of field supervisor, medical laboratory technologist, laboratory technician one each, and ten enumerators having nursing, public health or paramedics background. Their major responsibility was to fill out the questionnaires, carry out physical measurements and collect blood samples. The laboratory technicians were assigned for cold chain maintenance, sample processing, and the recording and reporting of biochemical measurements carried out using wet methods. Medical laboratory technologists were responsible for examining and verifying glucose levels and the value of the lipid profile including sending a 10% blood sample to the reference laboratory for external quality control.

A week-long training was organised in the two weeks prior to the beginning of data collection. The training was led by a STEPS team from WHO headquarters, Geneva and WHO SEARO, New Delhi. The local investigator team also joined the STEPS team as trainers. Prior to the training, the data collection team were oriented on the tools to be used to collect the data. Training focused on interview techniques, sampling process, household and individual selection, the use of the different kinds of templates and forms in the survey, the use and care of PDAs, a detailed explanation of the questionnaire and the technique to be used for physical measurements. The supervisors were also trained on downloading data from the PDAs as well as the troubleshooting of minor issues with the PDAs.

Overall, field operation and quality control was responsibility of field supervisor including coordinate with respective authorities at the field level, ensure completion of sampling frames, and select 20 households from each cluster. Also, the field supervisors were responsible for aggregating the data from individual PDAs to their laptop and forwarding them to the centre via email or by handing them over to the investigators.

### Definition of variables

The dependent variable in this analysis was occurrence of metabolic syndrome. The indicators analysed in this study are defined in Table 5. Two major predictor variables were included: individual characteristics: age in years (15–29, 30–44 and 45–69), gender (male and female), education (no formal schooling, primary, secondary and higher level), marital status (never married, currently married and divorced/widowed/separated) and caste/ethnicity, and community characteristics: ecological zone (mountain, hill and terai/plains) and place of residence (urban and rural) in this analysis.

**Table 5:** Showing the definition of variables used in the study.

Variable	Definition/measurements
Physical measurements	Height and weight were measured and body mass index (BMI) calculated as weight (kg.)/height (m^2^). Portable standard stature scale was used to measure height. Footwear (shoes, slippers, sandals) and hat were removed while measuring height. Respondents stood on a flat surface facing the interviewer with their feet together and heels against the backboard with knees straight. They were asked to look straight ahead and not tilt their head up, making sure that their eyes were at the same level as their ears. Height was recorded in centimetres. Weight was measured with a portable digital weighing scale (Seca, Germany). The instrument was placed on a firm, flat surface. Participants were requested to remove their footwear and socks, wear light clothes, stand on the scale with one foot on each side of the scale, face forward, place arms at their side and wait until asked to step off. Waist and hip circumference was measured using constant tension tapes (Seca, Germany) in centimeters^37^.
Blood Pressure measurements	Blood pressure was measured with a digital, automated blood pressure monitor (OMRON digital device) with medium cuff. Before taking the measurements, participants were asked to sit quietly and rest for 15 minutes with legs uncrossed. Three readings of the systolic and diastolic blood pressure were obtained using standard protocol. Participants rested for three minutes between each reading. The mean of the second and third readings was calculated. High blood pressure was defined as having systolic blood pressure ≥130 mm Hg and/or diastolic blood pressure ≥85 mm Hg during the study, or being previously diagnosed as having hypertension determined by sighting documentation such as a treatment record book or by the history of the participant taking medicine for high blood pressure.
Biochemical measurements	A separate mobile laboratory setting was used to collect biochemical data. The mobile laboratory contained all of the logistics and human resources required for the set up including a semi auto analyser and all of the chemicals required for blood glucose testing and lipid profile measurement. To ensure that the cold chain was maintained for the collected samples and for the preservation of the chemicals used for the tests, continuous electricity was ensured with an electric generator and refrigerator. Participants were instructed to fast overnight for 12 hours and diabetic patients on medication were reminded to bring their medicine/insulin with them and take their medicine after providing the blood sample to measure fasting glucose. Wet (liquid) method was used to measure blood lipids. A venous blood sample (4 ml of blood) was taken using a flashback needle with an aseptic technique and kept in plain and fluoride treated tubes. Those samples were kept in an ice pack carrier and brought to the mobile laboratory within an hour. Biochemical measurements of blood glucose and lipids were done using semi-automated procedures (Bioanalyzer, Analyticon, Germany) and commercially available kits (Analyticon, Germany). Plasma glucose was estimated using the GOD-PAP (glucose oxidase/peroxidise – phenol-4-amenophenazone) method. Serum triglycerides were estimated using the GPO-PAP (glycerol-3-phosphate oxidase/peroxidase-4-chlorophenol and 4-aminophenazone) method. HDL cholesterol was determined using the CHOD-PAP (cholesteroloxidase/peroxidase – 4-phenol-aminoantipyrine) method^36^. External quality control of these biochemical investigations was performed by sending 10% of the samples to the nearest reference laboratory with standardized fully-automated procedures for biochemical measurement.
Metabolic syndrome [NCEP ATP III criteria]^38^	Any three of the five criteria below: • Waist circumference: >102 cm (Male), >88 cm (Female). • Fasting glucose ≥100 mg/dl or Rx. • Triglycerides ≥150 mg/dl or Rx. • HDL cholesterol: <40 mg/dl (Male), <50 mg/dl (Female); or Rx ≥130 mmHg systolic or ≥85 mmHg diastolic or Rx.
Metabolic syndrome [IDF criteria]^38^	Central obesity (waist circumference: ≥90 cm (Male), ≥80 cm (Female)) plus two of the following criteria below: • Fasting glucose ≥100 mg/dl or Rx • Triglycerides ≥150 mg/dl or Rx • HDL cholesterol: <40 mg/dl (Male), <50 mg/dl (Female); or Rx ≥130 mmHg systolic or ≥85 mmHg diastolic or Rx
Current smoking	Those who smoked in the past 30 days were considered as a current smoker.
Harmful Alcohol consumption	Detailed information on number of standard drinks consumed and frequency of consuming standard drinks in the last 30 days was obtained from current users. One standard drinks was considered as 10 grams of ethanol, the number of standard drinks was calculated using the amount consumed by participants.
Insufficient fruits and vegetable intake	Information on fruit and vegetables consumption in a typical week. Also, the number of servings of fruit and vegetables consumed on average per day. The amount of fruit and vegetables was measured using pictorial show cards and measuring cups (one standard serving of fruit or vegetables equals 80 grams).
Low Physical activity	Global Physical Activity Questionnaire (GPAQ) was used to assess physical activities^39^. The GPAQ asks participants about activities for vigorous and moderate activity at work, and vigorous and moderate activity in leisure time and time spent sitting. Culturally relevant Show-cards with examples were used to classifying activities. Physical activity related to transport and recreation and time spent in sedentary behaviour were also assessed. Physical activity related to transport included travel to work or market by walking or using a bicycle. Recreational activity included two types of activities based on severity, i.e., vigorous and moderate. Vigorous recreational activity was defined as any recreational activity that causes a large increase in heart rate and breathing; for example, games such as football, fast swimming and rapid cycling. Ten minutes of such activity was considered as involvement in vigorous recreational activity. Moderate recreational activity was defined as any kind of recreational activity that causes a moderate increase in heart rate and breathing; examples include yoga and playing basketball. Sedentary behaviour was defined as a behaviour where an individual spends time sitting at a desk, sitting with friends, travelling in a car, bus or train, reading a book, and so on. Analysis and categorization followed existing guidelines^40,41^ and the low physical activities were categorized to those who did not meet the criteria for vigorous and moderate intensity activities.
Abnormal waist to hip ratio^42^	Abnormal waist to hip ratio is defined as a waist–hip ratio >0.90 for males and >0.85 for females.

### Data processing and analysis

In order to obtain nationally representative estimates, the sampling weights were used. Chi-square statistics was used to assess the difference in risk factors by gender. To reflect clustering within individuals, we considered the number of risk factors that each participant had at the time of the survey (from 0 to 5) and examined the mean number and 95%CIs of risk factors by covariates. We examined the independent effects of covariates on risk factor clustering within individuals by modeling a multivariate Poisson regression model, with the number of risk factors as the dependent variable. All the analysis carried out was using complex survey design; wards were considered as cluster and ecological zones as strata.

Univariate and multivariate logistic regression were used to test associations between predictors and outcome variables using Stata SE 14. Adjusted odd ratio (AOR) was calculated using multiple logistic regression, with all predictors (age, gender, education, marital status, ecological zone and place of residence) included simultaneously in the model in order to assess the predictors of MetS. A p-value < 0.05 was considered as statistically significant.

### Ethical Considerations

This study was approved by the ethical review board of the Nepal Health Research Council. An informed written consent was obtained from all the participants. For the participants under the age of 18 years, informed consent from parent/legal guardian was obtained. Waste generated during the laboratory procedures was properly disinfected using aseptic techniques and safely disposed as per protocol. Blood samples were discarded after the biochemical measurements.
